# Theoretically optimal forms for very long-span bridges under gravity loading

**DOI:** 10.1098/rspa.2017.0726

**Published:** 2018-09-19

**Authors:** Helen E. Fairclough, Matthew Gilbert, Aleksey V. Pichugin, Andy Tyas, Ian Firth

**Affiliations:** 1 Department of Civil and Structural Engineering, University of Sheffield, Mappin Street, Sheffield S1 3JD, UK; 2 Department of Mathematics, CEDPS, Brunel University London, Uxbridge UB8 3PH, UK; 3 COWI UK Ltd, Bevis Marks House, Bevis Marks, London EC3A 7JB, UK

**Keywords:** bridges, catenary of equal strength, structural optimization, layout optimization

## Abstract

Long-span bridges have traditionally employed suspension or cable-stayed forms, comprising vertical pylons and networks of cables supporting a bridge deck. However, the optimality of such forms over very long spans appears never to have been rigorously assessed, and the theoretically optimal form for a given span carrying gravity loading has remained unknown. To address this we here describe a new numerical layout optimization procedure capable of intrinsically modelling the self-weight of the constituent structural elements, and use this to identify the form requiring the minimum volume of material for a given span. The bridge forms identified are complex and differ markedly to traditional suspension and cable-stayed bridge forms. Simplified variants incorporating split pylons are also presented. Although these would still be challenging to construct in practice, a benefit is that they are capable of spanning much greater distances for a given volume of material than traditional suspension and cable-stayed forms employing vertical pylons, particularly when very long spans (e.g. over 2 km) are involved.

## 1. Introduction

Since construction of the 137 m span Union bridge on the England–Scotland border in 1820, the world's longest bridge span has doubled approximately every 50 years, and nine out of the 10 longest bridge spans in history have been constructed in the last 20 years. In recent years, plans have been developed for bridges in Italy, Norway and Indonesia with spans of in excess of 3 km, while a more speculative, though still potentially feasible, proposal has been mooted for a bridge with 5 km spans over the Strait of Gibraltar. While these specific structures face challenges of a technical as well as economic and political nature, it is likely that bridge spans will continue to increase in the twenty-first century.

Forms for long-span bridges have evolved over several centuries. The modern suspension bridge form, pioneered by James Finlay in the USA, started to find favour at the turn of the nineteenth century [1], and is still employed in the world's longest span bridge structures, such as the 1991 m span Akashi Kaikyo Bridge in Japan [2]. In the past few decades, the ease and speed of construction of cable-stayed bridge forms has led to an increase in their popularity, particularly for spans up to approximately 1200 m. This development has led to debate about the relative merits of suspension versus cable-stayed bridges [3]. However, debate has often centred on the importance of various practical considerations, for example, relating to ease of construction or susceptibility to dynamic effects, rather than on a comparison of the theoretical efficiency of these forms. Furthermore, and most significantly, it has seldom been questioned whether these forms are appropriate when very long spans are involved, or whether more optimal structural forms exist. This is important as a non-optimal form will consume more material than is necessary, in some cases considerably more. Also, it is important to bear in mind that for any given material the form will limit the overall span that can be attained. In the case of the Akashi Kaikyo Bridge, consultant M. Ito stated that high-strength steel wire was developed to reduce the dead weight *because more than 90 percent of the cross section of the main cables is used to carry the bridge's own weight* [4]. Similarly, K. H. Ostenfeld, Project Director for the Great Belt (East) Bridge with the longest span in the world prior to Akashi Kaikyo at 1624 m, has stated that one of two limiting factors for long-span bridges is *the limits for known materials in carrying their own weight* [5], governed by the well-known square-cube law [6]. Also Lewis [7] indicates a practical limiting span of less than 5 km for a suspension bridge when using steel, suggesting that materials with a higher specific strength (the strength/weight ratio) might be necessary to achieve longer spans. However, although lightweight composite materials [8,9], and even carbon nanotubes [10], have been proposed for long-span bridges, metals such as steel have advantages, such as far greater fracture toughness [11]. This coupled with plentiful supply and low cost means that steel is likely to remain the dominant material for suspension cables for the foreseeable future. Thus, while materials are unlikely to revolutionize longer span bridges in the near term, and recognizing that constructability factors still dominate consideration of economical solutions, there is the possibility for new, more materially efficient, structural forms to do so. This is the focus of the present study.

Although there have been attempts to identify improved designs for very long-span bridges by using engineering intuition [12,13], an alternative is to use layout optimization, the theoretical basis of which was developed by Michell [14] in the early twentieth century, building on earlier work by Clerk Maxwell [15]. This theory later stimulated the development of computer-based numerical layout optimization methods [16,17]. Modern numerical layout optimization methods have been found to be capable of identifying new forms and of obtaining very close estimates of the volumes of the corresponding exact solutions. For example, layout optimization was used to show that the minimum volume structure to carry a uniformly distributed load between pinned supports was not the parabolic arch (or cable) form which had presumed to be optimal since the time of Christiaan Huygens [18], but instead a more complex form comprising networks of orthogonal tensile and compressive elements near the supports and a central parabolic section; in this case the numerical volume obtained via extrapolation techniques was later shown to be within 0.001% of the exact analytical solution subsequently derived [19]. Layout optimization has also been used to investigate bridge-like forms, for example being used to show that changing the ratio of the limiting compressive to tensile material strength gives rise to a family of optimal structures which range from arch to cable-stayed forms [20], prompting follow on analytical studies by practitioners [21].

However, long-span bridge structures are dominated by self-weight and standard layout optimization techniques are not suitable in this case, since phenomena such as cable sag are not modelled intrinsically as part of the formulation. Simplified models which assume self-weight to be ‘lumped’ at end-nodes, and which implicitly assume that bending can be carried by the element between nodes [22] are problematic in cases where self-weight effects are significant. This is because in such a formulation the flexural effect of self-weight on the element itself is ignored, and physically meaningful solutions may not be generated (e.g. a solution might comprise long-spanning straight bars of constant cross section, which transmit a large proportion of their self-weight directly to supports).

In fact, when self-weight is taken into account it has been known for almost two centuries that each (non-vertical) element in an optimal structure must take the form of a *catenary of equal strength* [23,24]. This is an element which is free of bending and has a cross section which varies along its length, thus ensuring no excess material is present, a requirement in a rigorously optimal solution (notwithstanding that uniform cross sections are normally preferred in practice, for practical reasons). A key feature of such an element is that if the spatial positions of its end points are known in advance then that element can take up one of only two possible shapes, depending only on whether the force to be carried is compressive or tensile. This is significant as it means that the standard layout optimization procedure [16,17] can be modified such that every potential (non-vertical) member is an equal stress catenary element with an *a priori* defined shape. Thus straight elements are replaced with suitably curved compression and/or tension elements. This also means that applications where self-weight effects are significant can be tackled, such as very long-span bridges, enabling new reference solutions for bridge structures subjected to gravity loadings to be obtained.

In this paper, the formulation for elements of equal strength will first be outlined and then used to provide a new layout optimization formulation. This will then be used to determine the theoretically optimal form for a very long-span bridge of given span carrying gravity loading, with the volume of material required compared with that required to construct traditional bridge forms of the same span. Note that the influence of wind and other dynamic effects, while significant in the design and construction of such spans, are beyond the scope of the present study, although they can, in principle, be incorporated into structural optimization schemes.

## 2. Elements of equal strength

### (a) Catenary elements

Gilbert [23] developed relations to describe the shape of the *catenary of equal strength,* a structural form transferring its self-weight to two level end points. Its cross-sectional area *A* is proportional to the axial force *F*, hence its weight per unit length is *κF*, where *κ* is a proportionality constant. In an optimal skeletal structure the stresses at all points in an element must be purely axial, and equal to the value of the limiting material stress. For a material with unit weight *ρg* then the weight per unit length is *Aρg*. If the limiting stress in tension and compression is *σ*_T_ and *σ*_C_, respectively, then *F* = *Aσ*_T_ and *κ* = *ρg*/*σ*_T_ for tensile elements, and *F* =  − *Aσ*_C_ and *κ* = − *ρg*/*σ*_C_ for compressive elements. Unless otherwise stated, in this paper the limiting material stress will be taken to be the same in tension and compression, such that *σ*_T_ = *σ*_C_ = *σ*_0_.

Routh [25, Art. 453] provides a concise derivation of the equation for this catenary in Cartesian coordinates, which for our purposes can be reformulated as

2.1 $$\kappa y=\log(\cos(C_{1}-\kappa x))+C_{2}.$$

**Figure 1.**
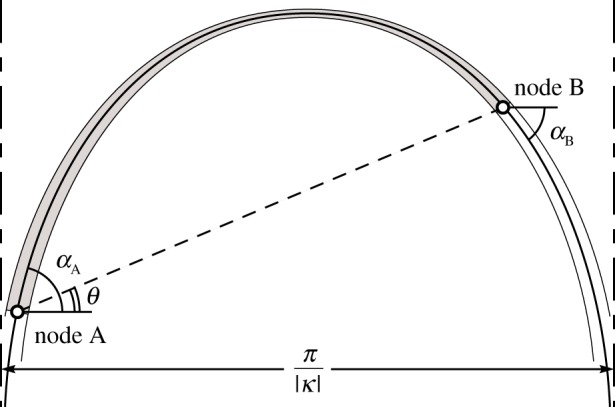
Approximate shape of an equally stressed element AB in compression.

If the origin is placed at the highest point of the curve *C*_1_ = *C*_2_ = 0. A plot of the curve is shown in figure 1. The curve has vertical asymptotes at ± *π*/2|*κ*|. Therefore, elements with a span of *π*/|*κ*| are not possible, as they would have infinite length and volume. (For the steel material used in this study, unless stated otherwise, the limiting material stress *σ*_0_ has been taken as 500 MPa and the unit weight *ρg* as 80 kN m^−3^, so that the maximum span of an element is approx. 20 km.)

We will need relations for elements connecting two arbitrary points on a plane, allowing these to be incorporated in the layout optimization formulation that will be described in §3. It can be shown, figure 1, that an equally stressed element connecting any two arbitrary points will consist of a segment of the curve defined by equation (2.1) with non-zero *C*_1_ and *C*_2_. If the coordinates of two nodes are denoted by (*x*_A_, *y*_A_) and (*x*_B_, *y*_B_), the expressions for the associated constants *C*_1_ and *C*_2_ can be established to be in the form

2.2 $$C_{1}=\kappa x_{A}-\arctan\left(\frac{\cos(\kappa (x_{B}-x_{A}))-e^{\kappa (y_{B}-y_{A})}}{\sin(\kappa (x_{B}-x_{A}))}\right)$$

and

2.3 $$C_{2}=\kappa y_{A}-\log\left(\cos\left(\arctan\left(\frac{\cos(\kappa (x_{B}-x_{A}))-e^{\kappa (y_{B}-y_{A})}}{\sin(\kappa (x_{B}-x_{A}))}\right)\right)\right).$$

The parameters that will be required in the layout optimization formulation are the reaction forces exerted on the element end points, and the volume of the element. Note also that the horizontal distance between the two connected points must be below *π*/|*κ*| in order for it to be possible to connect them; thus elements with a span greater or equal to this limit will not be added to the ground structure in the layout optimization formulation.

#### (i) Reaction forces

The angle of inclination of the centreline of the element at any point is given by

2.4 $$\alpha =C_{1}-\kappa x.$$

In order to produce purely axial stresses the force must also be inclined:

2.5 $$q_{y}=q_{x}\tan(\alpha ),$$

where *q*_*y*_ and *q*_*x*_ are the vertical and horizontal components of the force *q* at the given point. Component *q*_*x*_ will be constant over the length of the element as only vertical self-weight forces are applied between the end points. Given that an external force *q* is to be transmitted directly between the end points, *q*_*x*_ = *q*cos(*θ*), where *θ* is as shown in figure 1. Combining this with equation (2.5), gives

2.6 $$q_{y}=q\cos(\theta )\tan(\alpha ),$$

which, when used with values of *α*_A_ and *α*_B_ from equation (2.4), allows the vertical reaction forces *q*_*y*_ to be determined at the supports. Note that when self-weight effects become negligible, *α* will tend to *θ* and hence *q*_*y*_ in equation (2.6) will tend to *q*sin(*θ*).

#### (ii) Volume

The volume *V* of an element AB with unit width is given by

2.7 $$V_{\mathrm{AB}}=\int_{A}^{B}A\;ds,$$

where *A* is the cross-sectional area of the element at a given position along the element. By noting that the cross-sectional area at a point is proportional to the force at that point, it can be shown that

2.8 $$V_{\mathrm{AB}}=-\frac{q_{x}}{\rho g}(\tan(\alpha_{B})-\tan(\alpha_{A}))=-\frac{q\cos(\theta )}{\rho g}(\tan(\alpha_{B})-\tan(\alpha_{A})).$$

Note that there will be two sets of values for *α*_A_ and *α*_B_, one set for tensile elements and one set for compressive elements (see equation (2.4), noting that the sign of *κ* changes depending on whether tensile or compressive elements are involved).

### (b) Vertical elements

The equations presented in the preceding section cannot be applied to perfectly vertical elements as the curve never becomes completely vertical. Complementary equations for the thickness of an optimal vertical element of varying cross section are therefore now derived.

**Figure 2.**
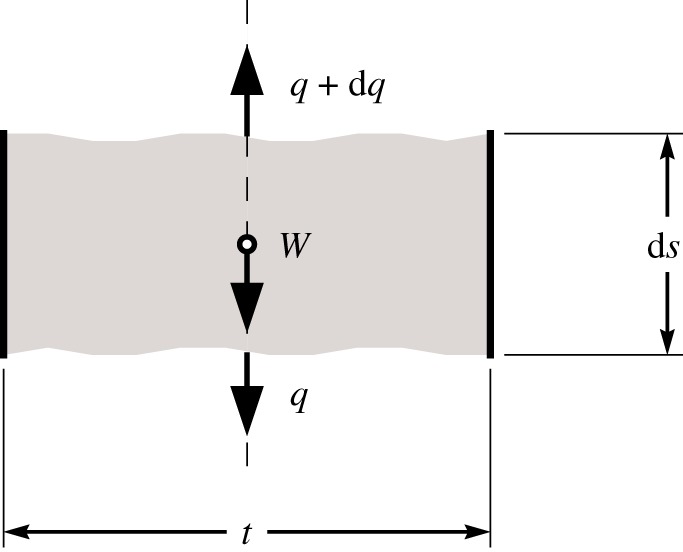
Forces acting on an infinitesimal slice of a vertical, equally stressed element.

If an infinitesimal slice of a vertical element is considered, as shown in figure 2, vertical equilibrium and the requirement that the stress is always equal to the limiting stress gives

2.9 $$-\frac{1}{q}\;dq=\kappa \;ds.$$

Integrating both sides between the endpoints A and B gives:

2.10 $$q_{B}=q_{A}\exp(\kappa (y_{A}-y_{B})).$$

#### (i) Reaction forces

Equation (2.10) relates the forces at each end of the element; however, unlike the catenary form, there is no value *q* which relates this to the force to be transmitted. Therefore, the reaction forces at the ends of vertical elements are defined in terms of *q*_A_. Trivially, the vertical reaction force at point A is defined as 1 × *q*_A_ and equation (2.10) provides the definition of the vertical reaction at point B. Also, since the element is vertical, *q*_A*x*_ = *q*_B*x*_ = 0.

#### (ii) Volume

The volume of the vertical element is found by integrating the cross-sectional area of the element over its length, which, due to (2.10), yields

2.11 $$V_{\mathrm{AB}}=\int_{A}^{B}A\;ds=\frac{q_{A}}{\rho g}\left(\exp\left(\kappa (y_{A}-y_{B})\right)-1\right).$$

## 3. Layout optimization

### (a) Standard formulation

**Figure 3.**
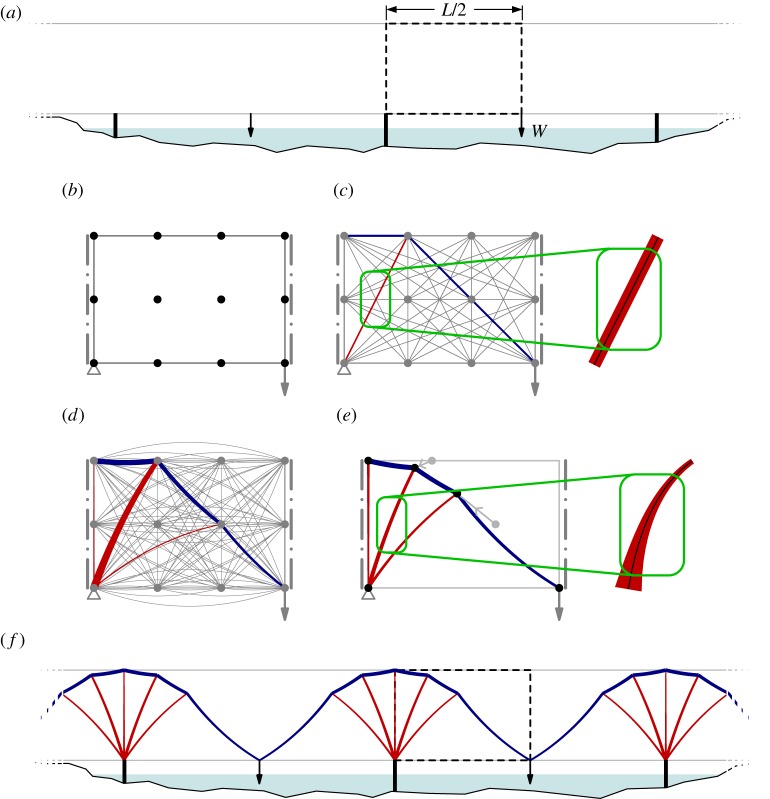
Procedure employed to identify optimal (minimum volume) bridge structures. Steps (*a*–*c*) describe the standard layout optimization formulation while steps (*a*,*b*) and (*d*) describe the new layout optimization formulation, used when self-weight effects are significant. Steps (*e*,*f* ) show the result of subsequently applying geometry optimization. (*a*) Problem definition (simplified multi-span bridge problem with multiple spans, with point load applied at the centre of each span; the highlighted half span can be modelled due to symmetry). (*b*) Design domain discretized with nodes and assumed boundary and load conditions. (*c*) Layout of straight (weightless) elements identified via layout optimization. (Compressive and tensile elements shown in red and blue, respectively.) (*d*) Layout of curved elements identified via layout optimization. (Curved elements may lie slightly outside the domain.) (*e*) Improved design, obtained by adjusting the positions of active nodes using geometry optimization. (Detail shows an element with exaggerated self-weight, demonstrating the curvature and non-uniform cross sections of these elements.) (*f* ) Resulting optimized design for the problem. (Online version in colour.)

The standard (weightless) numerical layout optimization procedure involves discretizing a design domain with *n* nodes, usually positioned on a uniform grid. These nodes are interconnected with *m* potential truss elements, forming a ‘ground structure’, and optimization is then used to find the minimum volume truss structure satisfying force equilibrium conditions, explained diagramatically in figure 3*a*–*c*.

The classical ‘equilibrium’ plastic truss layout optimization formulation for a single load case is defined in equation (3.1) as follows:

$$\min V=c^{T}q,$$

subject to:

3.1 Bq=f,

q≥0,

where *V* is the total volume of the structure. In the standard formulation not involving self-weight **q**^T^ = {*q*^+^_1_, *q*^−^_1_, *q*^+^_2_, *q*^−^_2_, …, *q*^−^_*m*_}, and *q*^+^_*i*_, *q*^−^_*i*_ are the tensile and compressive internal forces in bar *i* (*i* = 1, …, *m*); **c**^T^ = {*l*_1_/*σ*_T_, *l*_1_/*σ*_C_, *l*_2_/*σ*_T_, *l*_2_/*σ*_C_, …, *l*_*m*_/*σ*_C_}, where *l*_*i*_ is the length of bar *i* and *σ*_T_ and *σ*_C_ are, respectively, the limiting material stress in tension and compression. Note that in the classical layout optimization formulation buckling instability is not modelled directly, though the specified limiting material stress in compression can if necessary be reduced to account for this. **B** is a suitable (2*n* × 2*m*) equilibrium matrix containing direction cosines and **f**^T^ = { *f*^*x*^_1_, *f*^*y*^_1_, *f*^*x*^_2_, *f*^*y*^_2_, …, *f*^*y*^_*n*_}, where *f*^*x*^_*j*_ and *f*^*y*^_*j*_ are the *x* and *y* components of the external load applied to node *j* ( *j* = 1, …, *n*). The presence of supports at nodes can be accounted for by omitting the relevant terms from **f**, together with the corresponding rows from **B**.

Consider now the contribution of a single element *i* to the volume of the structure and to the global equilibrium matrix, which can be written as *V*_*i*_ = **c**^T^_*i*_**q**_*i*_ and **B**_*i*_**q**_*i*_, where **c**_*i*_, **B**_*i*_ and **q**_*i*_ are, respectively, a vector containing terms that allow the volume to be derived from the element force, the local equilibrium matrix, and the vector containing element force terms. The relevant expressions can be written in expanded form as follows:

3.2 $$V_{i}=\left[\begin{array}{cc} \frac{l_{i}}{\sigma_{T}} & \frac{l_{i}}{\sigma_{C}} \end{array}\right]\;\left[\begin{array}{c} q_{i}^{+}\\ q_{i}^{-} \end{array}\right]$$

and

3.3 $$B_{i}q_{i}=\left[\begin{array}{cc} \cos\theta_{i} & -\cos\theta_{i}\\ \sin\theta_{i} & -\sin\theta_{i}\\ -\cos\theta_{i} & \cos\theta_{i}\\ -\sin\theta_{i} & \sin\theta_{i} \end{array}\right]\;\left[\begin{array}{c} q_{i}^{+}\\ q_{i}^{-} \end{array}\right],$$

where *θ*_*i*_ is the angle of inclination of the element.

This problem is in a form which can be solved using linear programming (LP), with the member forces in **q** being the LP variables. Although posed as a plastic design problem, when only a single load case is involved, as is the case here, the formulation furnishes solutions which are identical to those obtained when using an elastic (minimum compliance) formulation [22].

### (b) Formulation with elements of equal strength

To take account of the effects of self-weight, the standard formulation can be modified to include elements with equal strength along their length. Thus straight elements are replaced with suitably curved compression and/or tension members, as indicated in figure 3*d*. By using the relations given in §2, new terms for *V*_*i*_ and **B**_*i*_**q**_*i*_ can be established which take account of self-weight effects as follows.

#### (i) Inclined element with self-weight

The element volume and equilibrium expressions can be obtained using (2.8) and (2.6), respectively, and written in expanded form as follows:

3.4 $$V_{i}=\left[\begin{array}{cc} \frac{\cos(\theta_{i})\left[\tan\alpha_{A}^{+}-\tan\alpha_{B}^{+}\right]}{\rho g} & \frac{\cos(\theta_{i})\left[\tan\alpha_{B}^{-}-\tan\alpha_{A}^{-}\right]}{\rho g} \end{array}\right]\;\left[\begin{array}{c} {\hat{q}}_{i}^{+}\\ {\hat{q}}_{i}^{-} \end{array}\right]$$

and

3.5 $$B_{i}q_{i}=\left[\begin{array}{cc} \cos\theta_{i} & -\cos\theta_{i}\\ \cos\theta_{i}\tan\alpha_{A}^{+} & -\cos\theta_{i}\tan\alpha_{A}^{-}\\ -\cos\theta_{i} & \cos\theta_{i}\\ -\cos\theta_{i}\tan\alpha_{B}^{+} & \cos\theta_{i}\tan\alpha_{B}^{-} \end{array}\right]\;\left[\begin{array}{c} {\hat{q}}_{i}^{+}\\ {\hat{q}}_{i}^{-} \end{array}\right],$$

where *α*^+^_A_, *α*^−^_A_ and *α*^+^_B_, *α*^−^_B_ are the angles of inclination of the tensile and compressive equal stress catenary elements at points A and B, respectively, which are calculated from equation (2.4), and ${\hat{q}}_{i}^{+}$ and ${\hat{q}}_{i}^{-}$ are the tensile and compressive forces in the element (equivalent to *q*^+^_*i*_ and *q*^−^_*i*_ given in the previous section).

#### (ii) Vertical element with self-weight

In this case, the element volume and equilibrium expressions can be obtained using (2.11) and (2.10), respectively, and written in expanded form as follows:

3.6 $$V_{i}=\left[\begin{array}{cc} \frac{1-\exp\left[\kappa (y_{A}-y_{B})\right]}{\rho g} & \frac{\exp\left[\kappa (y_{A}-y_{B})\right]-1}{\rho g} \end{array}\right]\;\left[\begin{array}{c} {\hat{q}}_{i}^{+}\\ {\hat{q}}_{i}^{-} \end{array}\right]$$

and

3.7 $$B_{i}q_{i}=\left[\begin{array}{cc} 0 & 0\\ 1 & -1\\ 0 & 0\\ -\exp(\kappa (y_{A}-y_{B})) & \exp(\kappa (y_{A}-y_{B})) \end{array}\right]\;\left[\begin{array}{c} {\hat{q}}_{i}^{+}\\ {\hat{q}}_{i}^{-} \end{array}\right],$$

where in this case ${\hat{q}}_{i}^{+}$, ${\hat{q}}_{i}^{-}$ are tensile and compressive force components, equal to *q*_A_, the force exerted on node A.

It is entirely possible for the ground structure, and therefore the resulting solution, to contain any desired combination of weightless, inclined and vertical elements with self-weight.

### (c) Rationalization via geometry optimization

When using layout optimization the solution accuracy is controlled by the number of nodes used to discretize the design domain. If large numbers of nodes are employed then highly accurate numerical solutions can be obtained. However, the associated forms will often be complex, and also difficult to realize in practice. To address this a relatively coarse nodal discretization can instead be used to obtain a layout that can then be improved by adjusting the locations of nodes using the geometry optimization procedure described in [26], though with standard weightless bars replaced with elements of equal strength, using the relations described in the preceding section; sample results are shown in figure 3*e*,*f* .

## 4. Application to very long-span bridges

### (a) Problem definition

The modified layout optimization procedure can now be used to identify theoretically optimal forms for very long-span bridges. For the sake of simplicity it is here assumed that the central span of a notional multi-span bridge structure is being modelled, with the problem being as described in figure 3, but with the point loads *W* replaced with a uniformly distributed load *w*, applied at the same elevation. This configuration can also be used to approximately represent a bridge with a central main span and shorter side-spans. (In fact, this representation will be exact if the side-spans are equal to half the main span, and if reactions at the ends of the bridge are purely horizontal, as is approximately the case, e.g. for the Akashi Kaikyo Bridge.) However, other specific scenarios can readily be modelled using the general numerical procedure described. The magnitude of *w* is assumed to include both traffic loading and the self-weight of deck elements required to provide a continuous level traffic surface, though it does not include the self-weight of material required in the deck to carry axial forces, if present, which is automatically included in the model as part of the optimization process. Note also that in very long-span bridges traffic loading becomes less significant than the self-weight of the deck and cables, which means that the problem can justifiably be posed as a single load case layout optimization problem, furnishing a structural form which consumes the minimum possible volume of material to carry the distributed load *w* and the self-weight of the structural elements employed. (The inherent bending resistance of the pylons and deck is not considered in the optimization directly, but will be required to ensure that alternative load cases can be carried. Also, although wind and other dynamic effects are very important in cable-supported bridges, particularly when spans are long, various mitigation strategies can often be applied once the basic form has been established. For example, to counter wind-induced vibration it has recently been suggested that the use of slotted box girder decks will permit 5 km suspension bridge spans to be achieved [27], or alternatively active control systems can potentially be employed [28]. These are therefore not considered further here.)

In the interests of computational efficiency, a half span was modelled (taking advantage of symmetry) and the adaptive solution strategy developed by Gilbert & Tyas [17] was employed to enable problems with increasingly fine nodal resolutions to be solved. The load discretization strategy used in [18] was also employed in order to reduce the discretization error associated with the load. The largest model run contained 68 026 nodes and 2 313 734 325 potential connections, including overlaps (i.e. over 2 *billion* potential connections).

### (b) New reference volumes

Using the aforementioned assumptions, the layout optimization procedure has been used to obtain new reference solutions for a range of bridge span lengths *L*, obtained by using increasingly fine nodal resolutions; numerical results are shown in table 1. The spans quoted in the table assume that high-strength steel is employed for all elements, with a limiting material strength in tension and compression of 500 MPa and a unit weight of 80 kN m^−3^ - though other scenarios can readily be modelled using the general numerical procedure described. Also shown are extrapolated volumes computed using the power-law extrapolation scheme described in [18]. Models comprising *n*_*x*_ = 200, 240, …, 600 divisions were used to provide source data, where *n*_*x*_ is the number of nodal divisions across the full span (the number of nodal divisions in the height of the domain, *n*_*y*_ was taken as *n*_*x*_ × 3/8). A weighted nonlinear least-squares approach was used to find best-fit coefficient values for use in the extrapolation, with the weighting factor taken as *n*_*x*_, to increase the influence of fine resolution solutions.

**Table 1.** Numerical volumes ( × *wL*^2^/*σ*_0_) for various bridge spans and nodal resolutions.

		span			
	*n*_*x*_	0 km (weightless)	1 km (0.16(*σ*_0_/*ρg*))	2.5 km (0.4(*σ*_0_/*ρg*))	5 km (0.8(*σ*_0_/*ρg*))
numerical	200	0.726937	0.781744	0.875206	1.068504
	240	0.726759	0.781493	0.874886	1.067978
	280	0.726624	0.781327	0.874680	1.067679
	320	0.726535	0.781206	0.874535	1.067468
	360	0.726475	0.781120	0.874425	1.067293
	400	0.726425	0.781050	0.874337	1.067149
	440	0.726378	0.780995	0.874262	1.067038
	480	0.726342	0.780945	0.874201	1.066947
	520	0.726313	0.780903	0.874150	1.066866
	560	0.726286	0.780871	0.874109	1.066804
	600	0.726267	0.780844	0.874075	1.066753
	∞^a^	0.726031	*0.780531*	*0.873674*	*1.066152*
analytical	—	0.7260325^b^	—	—	—
diff. (%)	—	0.0002	—	—	—

Notes: The new reference solutions are shown in italics typeface. (Quoted span lengths assume a limiting material stress *σ*_0_ = 500 MPa and unit weight *ρg* = 80 kN m^−3^.)

^a^Obtained by extrapolation, using a power-law extrapolation scheme as used in [18].

^b^Analytical solution obtained by Pichugin *et al.* [20].

For the 0 km span (weightless) case the extrapolated value was found to compare closely with a recently published analytical value [20], showing that high-precision results can be obtained. For the other spans considered the extrapolated values furnish new reference volumes, providing benchmark values against which alternative bridge designs can be judged. (As suggested by Cox [29], just as there is a limit on the thermal efficiency of a heat engine, set by the Carnot cycle, so there is a lower limit on the volume of material necessary to form a structure. Even though this will not normally be achievable in practice, it provides a useful basis on which to judge alternative designs.)

### (c) New structural forms

**Figure 4.**
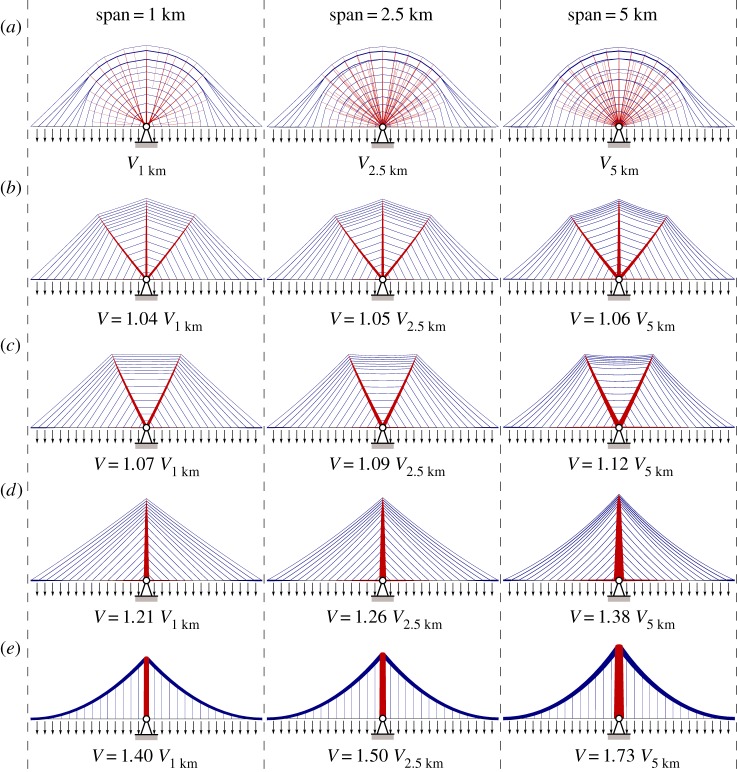
Bridge forms for various spans and associated volumes, expressed in terms of the reference volume, where *V*_1 km_ = 0.7805*wL*^2^/*σ*_0_, *V*_ 2.5 km_ = 0.8737*wL*^2^/*σ*_0_ and *V*_ 5 km_ = 1.0662*wL*^2^/*σ*_0_ (where *w* is the load applied at deck level, *L* is the span and *σ*_0_ is the limiting strength of the material). The three-span lengths considered, 0.16(*σ*_0_/*ρg*), 0.4(*σ*_0_/*ρg*) and 0.8(*σ*_0_/*ρg*), correspond to 1, 2.5 and 5 km, respectively, when *σ*_0_ is taken as 500 MPa and the unit weight *ρg* as 80 kN m^−3^. All solutions shown were optimized via geometry optimization. (*a*) Reference design (simplified layout shown for clarity). (*b*) Optimized triple split-pylon design. (*c*) Optimized double split-pylon design. (*d*) Optimized cable-stayed design. (*e*) Optimized suspension bridge design (all with variable cross-section cables). (Online version in colour.)

Structural forms corresponding to the new reference solutions for 1, 2.5 and 5 km span lengths are shown in figure 4*a*. To provide visually clear solutions a layout optimization discretization involving 30 nodal spacings across the full span was used prior to rationalizing the solutions using geometry optimization; see appendix A for details.

**Figure 5.**
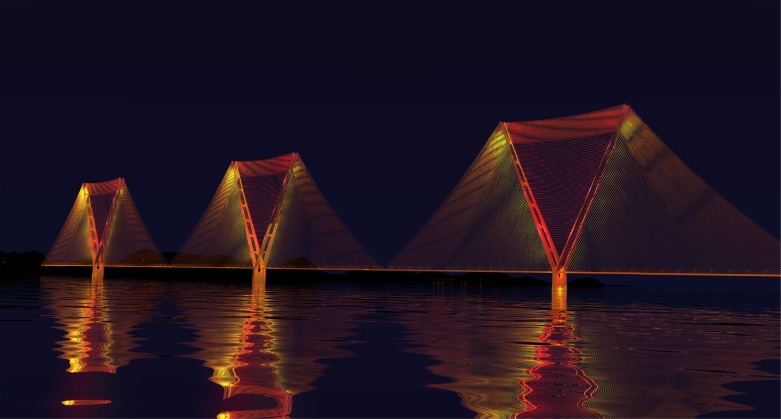
Split-pylon concept bridge to cross Strait of Gibraltar, with two 5 km main spans (not showing additional measures likely to be needed to counteract unbalanced live load effects, e.g. [30]). (Online version in colour.)

The new reference forms shown in figure 4*a* quite closely resemble the weightless Michell structure that was fully described analytically in [20], with tension and compressive elements aligned (near)orthogonally. They also include a series of inclined compressive elements radiating out from the supports, just as in [20]. The forms are also markedly different to traditional cable-stayed or suspension bridge forms, and would clearly be extremely difficult to construct in practice. However, to allow comparison with the former, the inclined compressive elements can readily be removed by omitting candidate inclined compressive elements when setting up the layout optimization problem. The resulting optimized cable-stayed bridge forms are shown in figure 4*d*, and differ slightly from the standard ‘harp’ cable arrangement in that they have non-uniform spacing of stays along the height of the pylon. However, most significantly, the consumed material is up to 38% greater than that required for the corresponding (near) optimal reference designs shown in figure 4*a*. Although the latter would be much more difficult to fabricate than the more conventional cable-stayed forms shown in figure 4*d*, they can be used to inspire a range of simplified forms. Thus figure 4*b*,*c* shows simplified split-pylon bridge structures comprising, respectively, three and two pylons, consuming, respectively, approximately 6 and 12% more material than the corresponding reference designs in the 5 km span case; a computer-generated render of a bridge comprising two 5 km spans designed to be potentially suitable for the hypothetical Strait of Gibraltar crossing is also shown in figure 5.

The optimized locations of all elements in the single, double and triple split-pylon cable-stayed structures shown in figure 4*b*–*d* were found using geometry optimization techniques (appendix A), indicating that a harp rather than fan style cable arrangement is most materially efficient.

Also, for comparative purposes, optimized suspension bridge configurations were obtained by applying geometry optimization to the standard suspension bridge layout, as shown in figure 4*e*. These bridges consume up to 73% more material than the corresponding reference designs shown in figure 4*a*. (The bridge structures described here are actually somewhat lighter than those found in constructed suspension bridges as the cable areas were here allowed to vary along the lengths of the cables.)

**Figure 6.**
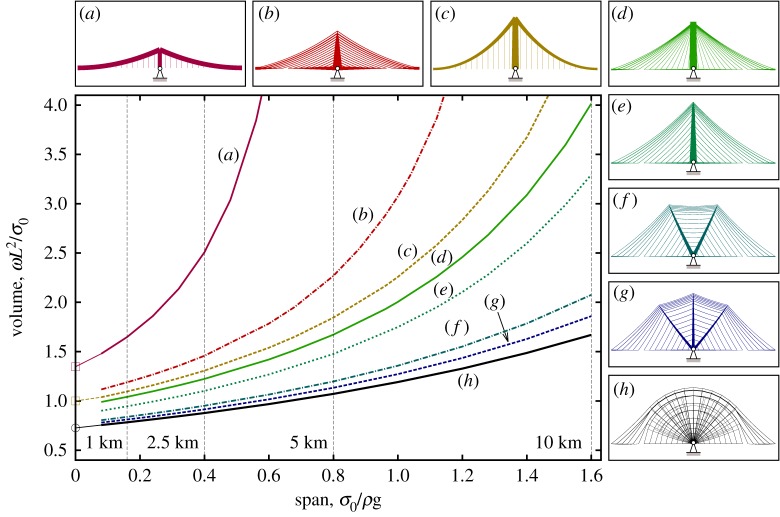
Volume versus span for various bridge forms, using equal stress catenary elements and with nodal positions identified via geometry optimization. (Span *L* = 0 data points are taken from the literature; see table 2 for details. Indicated span distances of 1, 2.5, 5 and 10 km assume that *σ*_0_ is taken as 500 MPa and the unit weight *ρg* as 80 kN m^−3^) (*a*) Suspension bridge, with span-to-dip ratio of 9, as in the Akashi Kaikyo Bridge, Japan. (*b*) Cable-stayed bridge with span-to-dip ratio of 4.4, as in the Russky Bridge, Russia (harp cable arrangement assumed here). (*c*) Suspension bridge with optimal pylon height. (*d*) Cable-stayed bridge with fan cable arrangement and optimal pylon height. (*e*) Cable-stayed bridge with optimal pylon height. (*f* ) Optimized double split-pylon design. (*g*) Optimized triple split-pylon design. (*h*) Reference bridge design. (Online version in colour.)

Figure 6 presents results for all the cases described in normalized form, covering spans of up to 10 km when using the same steel material as assumed before. Volumes for the fan style cable-stayed bridge form are also included for comparative purposes, along with those for cable-stayed and suspension bridge forms constrained to use the larger span-to-dip (*L*/*h*) ratios currently employed in the world's current longest cable-stayed and suspension bridge spans, respectively, found in the 1104 m span Russky Bridge, Russia (*L*/*h* = 4.4) and the 1991 m span Akashi Kaikyo Bridge, Japan (*L*/*h* = 9). (Traditional suspension bridges have generally been built with span-to-dip ratios of 8.5–13.5 and cable-stayed bridges with span-to-dip ratios of 3.4–4.9 [7].) It is evident that traditional bridge forms with larger span-to-dip ratios become very inefficient as spans increase, but that the simplified double and triple split-pylon forms remain comparatively efficient even when the span is long.

**Table 2.** Comparison of volumes ( × *wL*^2^/*σ*_0_) of traditional bridge forms with those of new reference solutions.

			span			
form	*L*/*h*^a^	figure	0 km (weightless)	1 km (0.16 (*σ*_0_/*ρg*))	2.5 km (0.4 (*σ*_0_/*ρg*))	5 km (0.8 (*σ*_0_/*ρg*))
suspension	9	6*a*,8*b*	1.347222 [31,32]	1.648	2.508	18.985
	optimal (4)	6*c*	1.000000 [31,32]	1.096	1.307	1.845
cable-stayed (fan)	4.4	7c		1.048	1.244	1.781
	optimal	6*d*		1.041	1.224	1.672
cable-stayed (harp)	4.4	6*b*	1.052273 [31]	1.189	1.458	2.269
	$\mathrm{optimal}\;(4/\sqrt{3})$	—	0.866025 [31]	0.954	1.112	1.492
cable-stayed (optimal)	4.4	*7d*		0.989	1.176	1.691
	optimal	6*e*		0.946	1.102	1.476
split-pylon (double)	9	8*e*		1.197	1.483	2.275
	4.4	7*e*		0.856	0.978	1.248
	optimal	*6f*		0.838	0.951	1.197
split-pylon (triple)	optimal	6*g*		0.811	0.914	1.134
reference	9	8*f*		1.130	1.320	1.711
	4.4	7*f*		0.817	0.921	1.137
	$\mathrm{optimal}\;(2\sqrt{2})$	6*h*, 7*g*, 8*g*	0.726031^[21]^	0.780531	0.873674	1.066152

Notes: A discretization of 30 nodal spacings across the full span and geometry optimization was used except for the optimal reference forms. Quoted span lengths assume a limiting material stress *σ*_0_ = 500 MPa and unit weight *ρg* = 80 kN m^−3^. The quoted volumes for the weightless designs are computed using explicit analytical expressions given in [31,32] (for suspension bridges), [31] (for cable-stayed bridges) and [20] (for optimal Michell structures).

^a^Optimal span-to-dip (*L*/*h*) ratios for weightless designs indicated in parenthesis, where available in the cited sources. (The optimal span-to-dip ratios for the longer span designs, which include self-weight, are similar, though not identical.)

### (d) Influence of span-to-dip ratio

**Figure 7.**
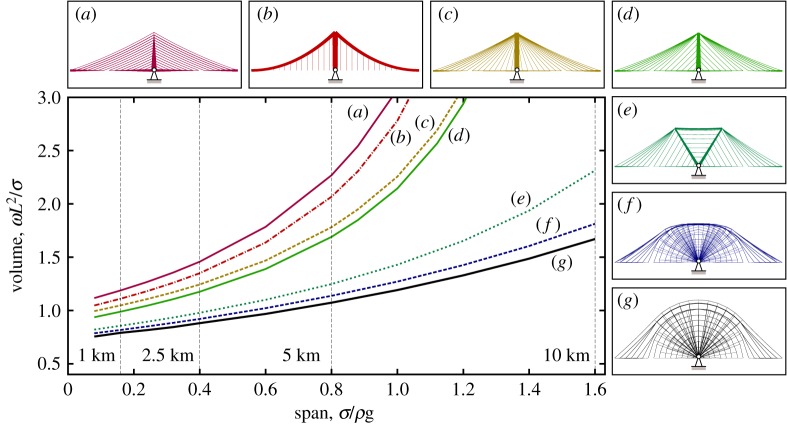
Span-to-dip ratio of 4.4-volume versus span for different bridge designs: (*a*) harp style cable-stayed; (*b*) suspension; (*c*) fan style cable-stayed; (*d*) optimized cable-stayed; (*e*) optimized double split-pylon; (*f* ) reference optimum form for the given span-to-dip ratio; (*g*) reference with unrestricted span-to-dip ratio for comparative purposes. (Online version in colour.)

**Figure 8.**
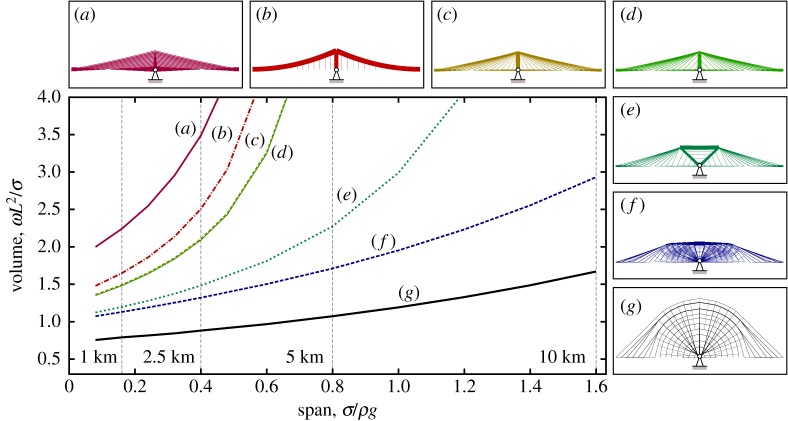
Span-to-dip ratio of 9-volume versus span for different bridge designs: (*a*) harp style cable-stayed; (*b*) suspension; (*c*) fan style cable-stayed; (*d*) optimized cable-stayed; (*e*) optimized double split-pylon; (*f* ) reference optimum form for the given span-to-dip ratio; (*g*) reference with unrestricted span-to-dip ratio for comparative purposes. (Online version in colour.)

The fact that a high span-to-dip ratio in a cable-supported bridge can lead to a high volume of material being required has been known for many years (indeed this prompted the seminal work of Davies Gilbert, who wanted to demonstrate to Thomas Telford that the initial shallow suspension bridge design for the Menai Straits was structurally inefficient [24]). However, a low span-to-dip ratio also requires the use of tall pylons, which can be problematic to construct. Also, when non-uniform loadings are present it has been observed that midspan deflections increase in suspension bridges when lower span-to-dip ratios are employed [30]. It is therefore of interest to explore the influence of the limiting span-to-dip ratio on the volume of material required, and to evaluate the performance of the new simplified forms when such limits are imposed. Figures 7 and 8 present volumes and corresponding forms when the span-to-dip ratios are limited to 4.4 and 9, respectively; key results from figures 6–8 are also tabulated in table 2, showing that the span-to-dip ratios identified as being optimal in the present study are considerably lower than those typically used in practice.

From figures 7 and 8 it is clear that even when a limit is placed on the span-to-dip ratio, the corresponding reference form consumes considerably less material than traditional forms. The simplified double split-pylon form is also much more materially efficient than traditional forms, suggesting that this latter form could prove popular in practice. When the span-to-dip ratio is increased to 9 the resulting double split-pylon form quite closely resembles the split-pylon design tentatively proposed by Starossek [13] some years ago, though the latter uses a fan style cable arrangement rather than the hybrid cable arrangement observed to be optimal here. (A small number of split-pylon cable-stayed bridges have been constructed in practice, though these have generally spanned relatively short distances, and aesthetic or specific practical reasons for their form have usually been cited by their designers. For example, the double split-pylon cable-stayed bridge constructed near Düsseldorf Airport in Germany in 2002 was so designed to keep the height of the structure low in order to avoid impeding air traffic [33]. Also, this bridge uses a fan style cable arrangement, rather than the hybrid cable arrangement found to be most materially efficient here.)

When the height available is unrestricted the optimal cable-stayed form resembles the harp style form but has a non-uniform spacing of cables up the pylon (figure 6); this also leads to a lower pylon height being required. As the height available is reduced the traditional harp style form (with uniform spacing of cables up the pylon) becomes less efficient than both the fan style form and also the suspension bridge form. Also, the optimal cable-stayed form begins to resemble the fan style form, and has a similar volume.

### (e) Influence of reducing the limiting compressive stress

**Figure 9.**
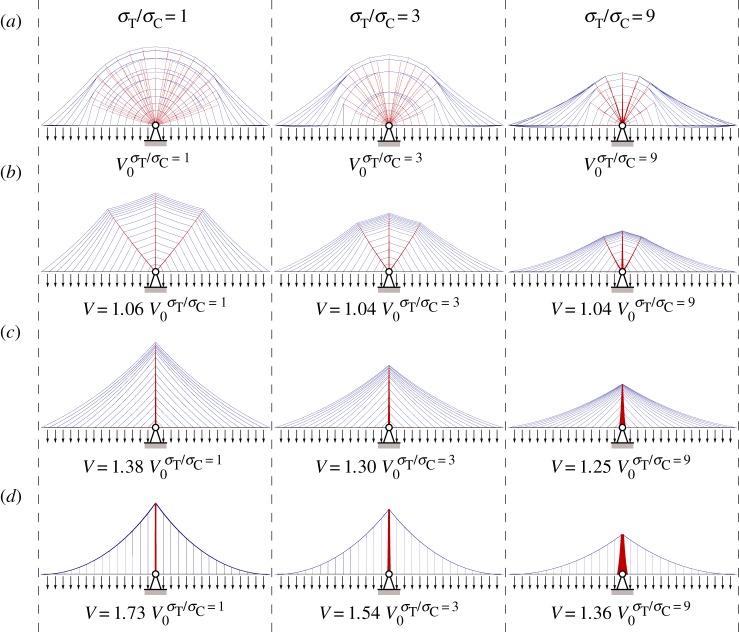
Effect of reducing the limiting compressive stress on selected forms shown in figure 4, for the span = 0.8(*σ*_0_/*ρg*) case (where the limiting tensile stress *σ*_ T_ = *σ*_0_ and the limiting compressive stress is reduced as indicated). Volumes shown are expressed in terms of the relevant reference volume. (*a*) Reference design (simplified layout shown for clarity). (*b*) Optimized triple split-pylon design. (*c*) Optimized cable-stayed design. (*d*) Optimized suspension bridge design. (All with variable cross-section cables.) (Online version in colour.)

In this study, the limiting material stress has thus far been assumed to be the same in tension and compression. However, it can be argued that a higher limiting stress should be adopted for tension elements, to account for the likelihood that higher strength steel will be used for the cables, and to account for a reduction in the stress sustainable by compression elements due to buckling effects. On the other hand, in recent years very high strength hot-rolled steel has become available which can potentially be used in the construction of pylon and deck elements carrying compressive stresses, and appropriate detailing can be used to try to mitigate the detrimental effects of buckling on the allowable stress. Nevertheless, it is of interest to explore cases when the limiting tensile stress *σ*_T_ exceeds the limiting compressive stress *σ*_C_. For example, figure 9 shows optimized bridge forms when the limiting stress in compression is reduced to one-third and then one-ninth of the limiting stress in tension (the latter case could e.g. represent a case where the limiting tensile stress is 1800 MPa and the limiting compressive stress is 200 MPa). Firstly, it is clear from the figure that the overall form of the optimal reference structure is largely unaffected, though the horizontal extent of the fan region over a support reduces (as is also the case when self-weight effects are neglected [20]). Also the optimal span-to-dip ratio increases for the reference structure, and also for all the other bridge forms considered. The relative volumes of the cable-stayed and suspension bridges fall, respectively, to 1.30 and 1.54 for the *σ*_T_/*σ*_C_ = 3 case, and to 1.25 and 1.36, respectively, for the *σ*_T_/*σ*_C_ = 9 case. This indicates that the advantage of the new forms over traditional forms remains, though does reduce somewhat when the limiting compressive stress is lower than the limiting tensile stress.

### (f) Commentary

This study has focused on identifying theoretically optimal forms for carrying the predominant load case for a hypothetical very long bridge span structure, i.e. self-weight loading, comprising the weight of the cables, pylons and deck, together with uniformly distributed traffic loading. It is sometimes argued that certain structural configurations become efficient when asymmetric loadings due to traffic are involved (e.g. considering the fan-style cable-stayed bridge configuration, the pylons are not subject to bending when asymmetric loadings due to traffic are involved, which is advantageous if traffic loads are high). However, in the case of very long-span bridges the magnitude of traffic loading in relation to self-weight becomes comparatively small, reducing this effect. For example, in studies recently undertaken by a bridge design consultancy for proposed suspension bridges incorporating spans ranging from 1.3 to 3.3 km, traffic loads contributed only between 15 and 23% of the loads carried by the main cables [34] (all loads unfactored). Thus although non-uniform traffic loading would need to be considered as part of the detailed design phase it is unlikely to change the overall findings of the present study when very long spans are involved. (By contrast when shorter spans are involved, of the sort likely to be constructed in the immediate future, non-uniform traffic loading can be expected to have an influence on form, with, in the case of multi-span bridges, alternate span traffic loading scenarios being particularly important. This, for example, led to a ‘crossing stay’ cable arrangement being chosen for the three-span Queensferry Crossing bridge across the Firth of Forth in Scotland, completed in 2017 [35]. Also, cable size may need to be increased to reduce deflections if these prove to be excessive.)

In this paper elements with non-uniform cross sections have been employed for all bridge forms considered, both new and traditional. This means that material in all cross sections is fully stressed, ensuring that the structures are as materially efficient as possible, thereby allowing reference solutions to be obtained. However, it should be noted that while methods of realizing non-uniform elements have been proposed (e.g. [36]), usually uniform cross sections are preferred in practice, which will slightly increase the overall weight of all bridges forms considered.

**Figure 10.**
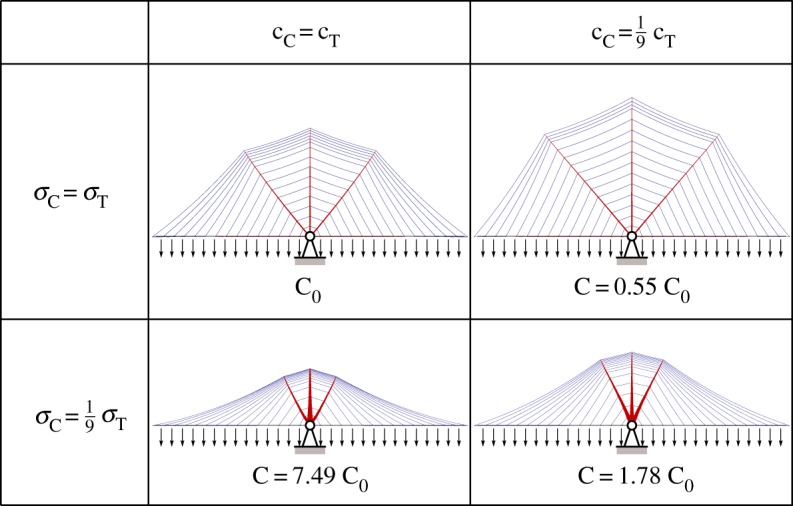
Effect of changing the relative cost of compressive and tensile members on the minimum cost *C* and corresponding geometry of the triple split-pylon bridge form considered in figure 9, where $C=\sum c_{T}V_{T}+\sum c_{C}V_{C}$, and where *c*_T_, *V*_T_ and c_C_,V_C_ are the cost coefficients and volumes for tensile and compressive members, respectively. (Two limiting tensile to compressive stress and cost ratios considered.) (Online version in colour.)

Although the present study has focused on efficiency, the method proposed can potentially be modified so as to minimize the likely cost of the overall bridge construction. For example, as the cost of cables is likely to be higher than the cost of compression elements, this can be accounted for by including suitable cost coefficients in the optimization objective function. For example, in figure 10 the minimum cost triple split-pylon design is sought, with the ratio of limiting tensile and compressive stress varied together with the relative cost of tensile and compressive members. Also, when modelling a bridge containing end spans, a cost could potentially be ascribed to the resultant horizontal reaction forces, which would in practice need to be carried by costly anchorages. These forces will generally be greatest in the case of suspension bridge forms, adding to their relative cost, though the other cable-supported forms considered herein would also require the use of external earth anchorages, due to the presence of tension at the supports. Although ‘self-anchored’ cable-stayed bridge designs have become popular due to ease of construction, a benefit of using earth anchorages for very long spans is that compression in the deck can be controlled; this is discussed further by Gimseng [37]. Alternatively boundary conditions in the optimization problem can easily be changed to, for example, prevent a solution being generated which requires transmission of horizontal forces to supports.

**Figure 11.**
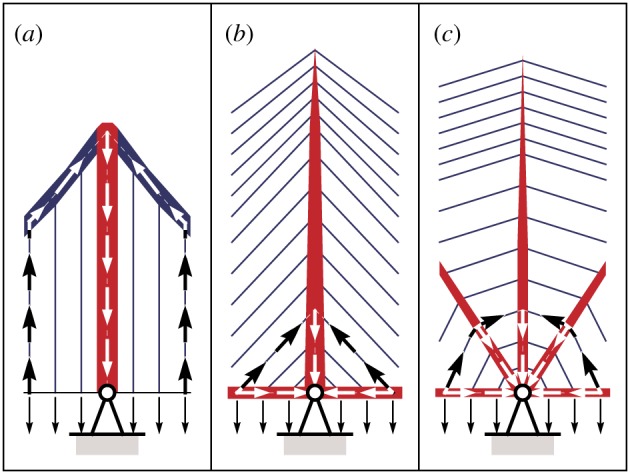
Appraising the relative efficiency of bridge forms by considering how deck loads are transmitted back to a support. (*a*) Suspension bridge: the load path is comparatively long, so a large volume of material is required to transmit the forces involved. (The same situation occurs in a fan cable-stayed bridge.) (*b*) Harp cable-stayed bridge: the load path is kept short, but acute angles between the elements lead to high cable forces, and also to significant induced forces in the deck. (*c*) Split-pylon cable-stayed bridge: the load path remains relatively short but the angles between the elements are less acute, ensuring cable and induced deck forces are lower. (Online version in colour.)

Finally, an explanation for the comparative efficiency of the split-pylon form is given in figure 11; this involves considering how directly deck loads are transmitted back to the supports. Although constructing very long-span bridges based on these latter designs would no doubt be challenging, the reward—significantly reduced material consumption—is clear.

## 5. Conclusion

A means of obtaining the theoretically optimal bridge form to span a given distance under the action of gravity loading has been described. This required the development of a new numerical layout optimization procedure capable of intrinsically modelling the self-weight of the constituent structural elements. To achieve this, equal strength catenary elements were employed, allowing phenomena such as cable sag to be modelled in the optimization process in an entirely natural manner. The procedure has been applied to the design of hypothetical very long-span bridges, enabling the optimal reference form and associated required volume of material to be established for a given span. The reference volume provides a benchmark against which traditional or proposed new bridge designs can be objectively judged.

It has been found that the reference bridge forms differ markedly to traditional suspension bridge and cable-stayed bridge forms involving vertical pylons, and also require much less material to construct, especially when very long spans are involved. For example, a conventional suspension bridge form spanning 5 km requires up to 73% more material than the corresponding reference design when using steel. The reference designs have also been used as inspiration for simple yet highly efficient bridge designs involving split pylons, which require comparatively little more material than the reference designs.

## Supplementary Material

Reference Solutions

## Supplementary Material

Geometry Optimized Solutions

## Figures and Tables

**Figure 12.**
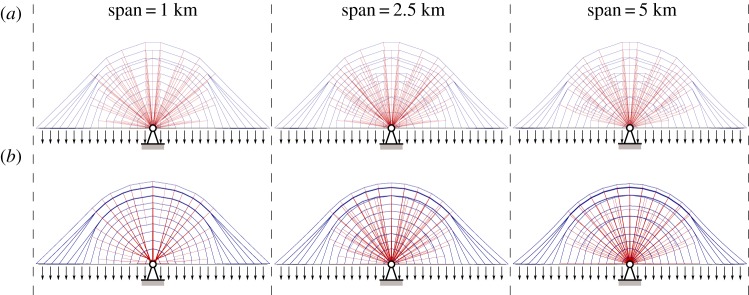
Bridge forms rationalized using geometry optimization. (*a*) Layout optimized solutions, obtained using a coarse nodal discretization. (*b*) Solutions obtained after using geometry optimization to rationalize (*a*). (Quoted span lengths assume a limiting material stress *σ*_0_ = 500 MPa and unit weight *ρg* = 80 kN m^−3^.) (Online version in colour.)

**Figure 13.**
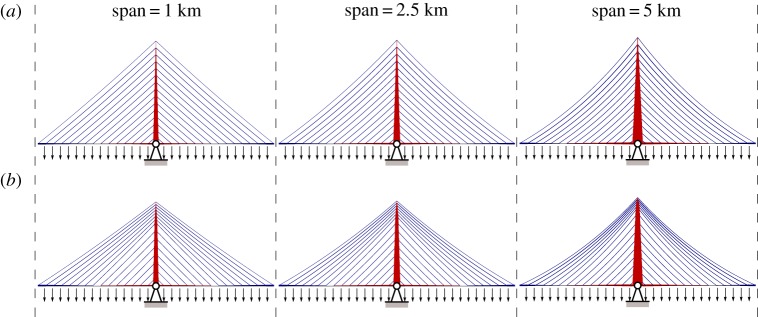
Sample bridge forms optimized using geometry optimization. (*a*) Harp style cable-stayed bridge, with cables intersecting the pylon at equal vertical intervals. (*b*) Modified form obtained after applying geometry optimization to the structure in (*a*), showing resulting optimized pylon/cable intersection locations. (Quoted span lengths assume a limiting material stress *σ*_0_ = 500 MPa and unit weight *ρg* = 80 kN m^−3^.) (Online version in colour.)

## Data accessibility

Geometrical data describing key reference and geometry optimized bridge forms are provided in the electronic supplementary material.

## Appendix A

Geometry optimization has been used to generate many of solutions presented. For example, figure 4*a* was obtained by starting with a layout optimized solution, obtained using a relatively coarse nodal discretization, and then using geometry optimization to rationalize the solution; see figure 12 for ‘before’ and ‘after’ layouts for this case. It is evident that the half-wheel-like section over the supports appears smoother and more clearly defined after geometry optimization has been performed.

Geometry optimization was also used to establish the most efficient joint positions for the traditional and new simplified split pylon bridge forms considered. For example, figure 13 shows ‘before’ and ‘after’ layouts for a harp style cable-stayed bridge configuration, which indicates that a lower volume solution is obtained if the cables are allowed to ‘bunch up’ near the top of the pylon. In each case a discretization of 30 nodal spacings across the full span was used to ensure visually clear solutions could be obtained (sensitivity studies indicate that this discretization gives solutions which are slightly above, though within approximately 0.1%, of the solutions which can be obtained using extremely fine numerical discretizations).
