# Supplementary figures and images for: Theoretically optimal forms for very long-span bridges under gravity loading
Source: Proc Math Phys Eng Sci. 2018 Sep 19;474(2217):20170726. doi: 10.1098/rspa.2017.0726 (PMC6189590; doi:10.1098/rspa.2017.0726)

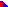

Supplement: Reference Solutions [file rspa20170726supp1.zip › Reference Solutions/1km_filtered.pdf]

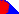

Supplement: Reference Solutions [file rspa20170726supp1.zip › Reference Solutions/2pt5km_filtered.pdf]

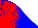

Supplement: Reference Solutions [file rspa20170726supp1.zip › Reference Solutions/5km_filtered.pdf]
